# The Effect of Transparent Conducting Oxide Films on WO_3_-Based Electrochromic Devices with Conducting Polymer Electrolytes

**DOI:** 10.3390/polym15010238

**Published:** 2023-01-03

**Authors:** Benedict Wen-Cheun Au, Kah-Yoong Chan, Gregory Soon How Thien, Mian-En Yeoh, Mohd Zainizan Sahdan, Hanabe Chowdappa Ananda Murthy

**Affiliations:** 1 Centre for Advanced Devices and Systems, Faculty of Engineering, Multimedia University, Persiaran Multimedia, Cyberjaya 63100, Malaysia; 2 Sri Desa International Secondary School, Taman Desa, Kuala Lumpur 58100, Malaysia; 3 Infineon Technologies (Kulim) Sdn. Bhd., Kulim Hi-Tech Park, Kulim 09090, Malaysia; 4 Faculty of Technical and Vocational Education (FPTV), Universiti Tun Hussein Onn Malaysia, Parit Raja, Batu Pahat 86400, Malaysia; 5 Department of Applied Chemistry, School of Applied Natural Science, Adama Science and Technology University, Adama P.O. Box 1888, Ethiopia; 6 Department of Prosthodontics, Saveetha Institute of Medical and Technical Science (SIMAT), Saveetha Dental College & Hospital, Saveetha University, Chennai 600077, Tamil Nadu, India

**Keywords:** electrochromic device, sol–gel, transparent conducting oxide, tungsten oxide, polypyrene

## Abstract

Over the past few decades, electrochromism has been a prominent topic in energy-saving applications, which is based on the mechanism of altering the optical transmittance of EC materials under the effect of a small applied voltage. Thus, tungsten oxide (WO_3_) is a significant chemical compound typically applied in electrochromic devices (ECDs) as it is responsible for the optical transmittance variation. In this work, the WO_3_ films were produced through a sol–gel spin-coating method. The effect of various transparent conducting oxides (TCOs, which are indium-doped tin oxide (ITO), fluorine-doped tin oxide (FTO) glass substrates, and aluminum-doped zinc oxide (AZO)) was investigated in the construction of ECDs. Based on a conducting polymer polypyrene carbonate electrolyte, ITO and aluminum-doped zinc oxide (AZO)-coated glasses were also examined as counter electrodes. The electrode combination employing FTO and ITO as the TCO and counter electrode, respectively, exhibited the most significant coloration efficiency of 72.53 cm^2^/C. It had coloring and bleaching transmittance of 14% and 56%, respectively, with a large optical modulation of 42%. In addition to that, ECDs with the AZO counter electrode have the advantage of lower intercalation charges compared to ITO and FTO. Hence, this research offers a new avenue for understanding the role of common TCO and counter electrodes in the development of WO_3_-based ECDs with conducting polymer electrolytes.

## 1. Introduction

Since the discovery of electrochromism by Deb in the 1970s [1], the phenomenon has gained widespread interest in energy-saving applications, such as smart windows [2], rear-view mirrors [3], and sensors [4]. Therefore, electrochromism is the phenomenon of reversible change in optical transmittance, to which a small electric field is applied [5]. Among the electrochromic (EC) materials, tungsten oxide (WO_3_) is one of the most studied chemical compounds owing to its distinctive properties, such as high coloration efficiency, excellent reversibility, quick production, and low production cost [6,7,8,9]. The general reversible color-changing mechanism of WO_3_ is typically utilized in the following equation [10]:

WO_3_ (colorless) + *xM*^+^ + *x*e^−^ ⇌ *M_x_*WO_3_ (blue) (1)

where *M* = H^+^, Li^+^, and Na^+^ and *x* is the ion concentration. A conjugated polymer component, such as polypyrene carbonate [11], polypyrrole [12], polyaniline [13], polythiophene [14], or polyethylene [15], is usually used in the fabrication of the electrochromic device (ECD). The formation of the conducting polymer electrolytes usually involves the addition of conducting electrolytes, such as KCl, LiClO_4_, K_2_SO_4_, and KNO_3_ [16]. Thus, the ions can travel in the conducting polymer electrolytes between both conducting electrodes to produce the coloration and bleaching processes. Under small voltage, the EC material changes color due to the simultaneous insertion of electrons and cations (Li^+^, H^+^, and Na^+^), which produces tungsten bronze [17]. Hence, the color change is due to the conversion in oxidation states from W^6+^ to W^5+^. Alternatively, the reversing process in the voltage polarity leads to the removal of cations, which subsequently bleaches the EC material [18].

Based on the fabrication methods of EC-WO_3_ films, multiple methods have been employed, such as magnetron sputtering [19], pulsed laser deposition [20], and the sol–gel route [21]. Nonetheless, most of the advanced fabrication routes of WO_3_ require sophisticated machinery or high-vacuum conditions that increase the risks involved with handling potentially dangerous compounds, such as lithium perchlorate, in the liquid polymer electrolyte solution [22]. Resultantly, the sol–gel route is often adopted for the fabrication of WO_3_ films due to its low operation cost, simplicity, and ability to fabricate large-area films with high coloration efficiency [23]. In terms of transparent conducting oxides (TCOs), the commonly used substrates in ECDs are indium-doped tin oxide (ITO) and fluorine-doped tin oxide (FTO)-coated glass substrates [24,25]. Nevertheless, the role of these TCOs, either as anodes or cathodes in ECDs, is not fully understood. The choice of TCOs can significantly affect the efficiency and production cost of ECDS in their commercialization potential. In general, SnO_2_-based TCOs are n-type semiconductors with electrical conductivity in the range of 10^4^ S/cm where this exceptionally low resistivity can be attained by n-type doping to a degenerate state in SnO_2_. This inevitably gives rise to the much-needed high n-type carrier concentration, in the range of 10^19^ to 10^20^ cm^−3^, which emerges from two shallow donors [26]. As opposed to the doped SnO_2_ species of conductive electrodes, AZO is another type of metal oxide material that has established itself as a member of the TCO family. It should be noted that AZO is a favorable replacement for ITO as a consequence of the scarcity and price of indium. On a side note, AZO is achieved by introducing aluminum atoms into zinc oxide (ZnO) [27]. Therefore, these TCOs should be studied on their EC performance and their interaction with the active layers and conducting polymer electrolytes.

In this study, WO_3_ films were fabricated via the sol–gel route spin-coating technique on ITO- and FTO-coated glasses as substrates. The fabricated WO_3_ films were assembled with lithium perchlorate–propylene carbonate electrolyte into ECD using ITO and aluminum-doped zinc oxide (AZO)-coated glasses as counter electrodes. It is worth mentioning that AZO is a potential alternative to ITO due to the scarcity and high cost of indium. Although ITO is superior in terms of electrical properties, zinc oxide is abundant in nature and a low-cost material where it has been adopted as TCO layers in recent years. The ECDs were characterized for their optical properties by using an ultraviolet-visible (UV-Vis) spectrophotometer, while EC properties were studied by using cyclic voltammetry (CV) and chronoamperometry (CA) methods.

## 2. Materials and Methods

### 2.1. Materials

Tungsten hexachloride (WCl_6_), hydrogen peroxide (H_2_O_2_), lithium perchlorate (LiClO_4_), and propylene carbonate (PC) were purchased from Sigma-Aldrich, Rockville, MD, USA. Glacial acetic acid (CH₃COOH) was obtained from BASF Chemical Company, CA, USA. Subsequently, absolute ethanol (C_2_H_6_O) was purchased from Merck, NJ, USA. For the TCOs, ITO and FTO were obtained from Han Xin Industry Co., Ltd., Shanghai, China. Meanwhile, AZO-coated glass slides were purchased from Photonik Lte. Ltd., Bukit Panjang Ring Road, Singapore. All chemicals utilized in this study were of analytical grade and were used as received without any further purification.

### 2.2. The Fabrication of WO_3_ Films on Different TCOs

The WO_3_ films in this work were fabricated via the sol–gel route spin-coating method. The WCl_6_ powder (99.99%) was applied as the W precursor, C_2_H_6_O (99.99%) was used as a solvent, CH_3_COOH (99.5%) was utilized as a chelating agent, and H_2_O_2_ 30% was used as a strong oxidizing agent. Prior to the spin-coating process, the ITO and FTO glass substrates were cleaned with deionized water, acetone, and isopropanol in an ultrasonic bath. For the sol–gel solution, 1 g of WCl_6_ powder was dissolved in 20 mL of absolute ethanol, and then 2 mL of CH_3_COOH was added to the solution. The solution was stirred for 30 min in an ambient environment before H_2_O_2_ was carefully added and stirred for another 30 min. Subsequently, the solution was continuously stirred for 2 h at 40 °C to yield a homogenous transparent solution. The solution was then spin-coated at 3000 rpm for 30 s before being heated on a hot plate at 100 °C for 3 min. Finally, the films were further annealed at 250 °C in an ambient environment.

### 2.3. The Study of the EC Properties of WO_3_-Based ECDs

The optical transmittances of the WO_3_ films were evaluated with an ultraviolet-visible (UV-Vis) spectrophotometer (Avantes AvaSpec-ULS2048CL, NS Apeldoorn, The Netherlands). The EC properties were measured by using the potentiostat-galvanostat setup (Metrohm Autolab PGSTAT204, Selangor, Malaysia) in a three-electrode configuration. Based on the three-electrode configuration, the WO_3_ films were used as the working electrode, the Ag/AgCl electrode was the reference electrode, and a platinum wire was the counter electrode. The liquid polymer electrolyte was produced with 1 M of LiClO_4_ in PC. Subsequently, a CV study was performed under an applied voltage bias from −3 V to 3 V with a scan rate of 100 mV/s for diffusion coefficient analysis. Furthermore, CA analysis was investigated to study the coloring and bleaching mechanics of the WO_3_ films. The coloring and bleaching transmittances were also recorded simultaneously in the wavelength range from 200 nm to 1000 nm. Lastly, the WO_3_ films deposited on ITO and FTO substrates were fabricated into ECDs using AZO and ITO as the counter electrodes. In this work, the ECDs of ITO/WO_3_/LiClO_4_-PC/AZO were named ECD(I:A), ITO/WO_3_/LiClO_4_-PC/ITO was named ECD(I:I), FTO/WO_3_/LiClO_4_-PC/AZO was ECD(F:A), and FTO/WO_3_/LiClO_4_-PC/ITO was ECD(F:I).

## 3. Results and Discussions

### 3.1. Structural Properties of WO_3_ Films with Various TCOs

**Figure 1:**
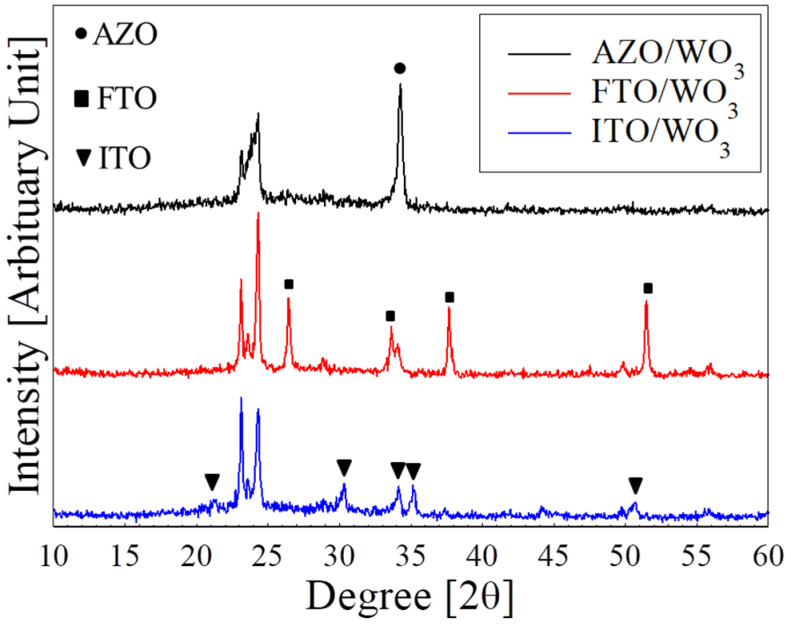
The XRD patterns of the as-deposited WO_3_ films on different TCO substrates.

In this study, the structural properties of the WO_3_ films deposited on different TCO substrates were investigated using XRD analysis (PANalytical X’Pert Pro MRD PW 3040/60, UK) (see Figure 1). By eliminating the peaks observed from the ITO, FTO, and AZO substrates (depicted by the symbols), the dominant characteristic of the diffraction peaks at 23.1°, 23.6°, and 24.3° was observed to correspond to the (002), (020), and (200) planes, respectively. Based on the peak positions, the monoclinic crystal structure of the WO_3_ films deposited on substrates was confirmed [10]. Consequently, the growth of WO_3_ films was confirmed, which was successfully deposited on ITO, FTO, and AZO substrates. Nonetheless, the FTO/WO_3_ film revealed higher peak intensity than ITO/WO_3_ and AZO/WO_3_. Moreover, this decrease in XRD peak intensity is an indication of a low degree of crystallinity. The role of different TCOs could have affected the spin-coating process of the WO_3_ film, which can be attributed to the stacking faults, dislocation, grain boundaries, or grain boundary scattering [28]. Thus, choosing a suitable TCO based on the desired WO_3_ crystallinity may aid in improving the performance of ECDs.

### 3.2. EC Performance of WO_3_-Based ECDs with Various TCOs

**Figure 2:**
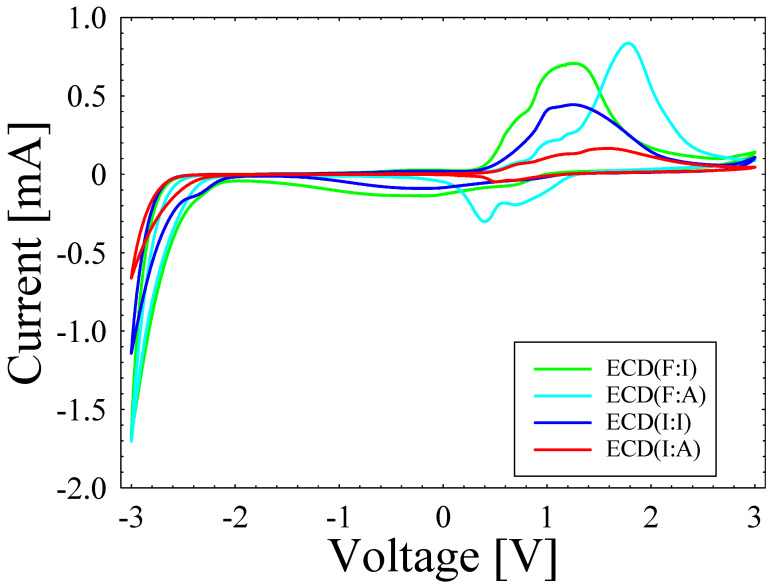
CV curves of ECDs with various combinations of transparent electrodes.

In the EC characteristic analysis, Figure 2 depicts the CV curves of ECD(I:A), ECD(I:I), ECD(F:A), and ECD(F:I) samples. Based on the curves, it can be seen that the highest anodic and cathodic peak currents were exhibited by ECD(F:A) with values of 0.834 and −1.705 mA, respectively. In addition, the electrochemical reaction increased within the ECD during the bleaching and coloring cycles, as indicated by the high anodic and cathodic peak currents [29]. Meanwhile, ECD(F:I) indicated relatively high anodic and cathodic peak currents of 0.706 and −1.665 mA, respectively. ECD(I:I) also presented an anodic peak current of 0.443 mA and a cathodic peak current of −1.142 mA. In terms of the lowest anodic and cathodic peak current, this was observed with ECD(I:A), which produced values of 0.164 and −0.662 mA, respectively.

To further understand the fabricated ECDs in liquid polymer electrolytes, the obtained anodic and cathodic peak currents were translated into diffusion coefficient values. Generally, the diffusion coefficient denotes the rate of ion movement in the electrolyte and is crucial to determining the extent of ECD performance. Hence, the Randle-Sevcik equation [21] was utilized in this study as follows:

*i* = 2.72 × 10^5^ × *n*^3/2^ × *D*^1/2^ × *C_o_* × *υ*^1/2^ (2)

where *i* is the peak current, *n* is the number of electrons, *D* is the diffusion coefficient, *C_o_* is the concentration of active ions in the electrolyte, and *υ* is the scan rate in the experiment. Therefore, the diffusion coefficient values of all four ECDs were summarized accordingly in Table 1.

**Figure 3:**
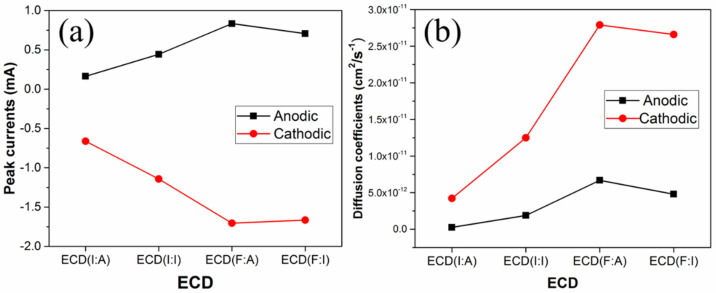
Graphs indicating the anodic and cathodic (**a**) peak currents and (**b**) diffusion coefficients of the ECDs.

Among the ECDs, the highest and lowest diffusion coefficients were observed in ECD(F:A) and ECD(I:A), respectively (see Figure 3). Hence, the low diffusion coefficients observed in ECD(I:A) were likely due to the increased sheet resistance in the utilized ITO substrate during the ITO/WO_3_ annealing process. Moreover, the increase in sheet resistance in ITO films was reported by Bhaskar et al. Their work further explained that oxygen plays an important role in increasing the resistivity of ITO films. As the primary conduction process mainly depends on oxygen vacancies (*V_o_*), the post-annealing process produces the incorporation of oxygen into the ITO films. Therefore, the reduction in the amount of *V_o_* indicates increased resistivity, which confirms the low diffusion coefficient [30]. Based on the FTO film results, the resistivity of the FTO films remains almost unchanged even at high annealing temperatures. Thus, this explains the larger CV curves and, subsequently, greater diffusion coefficient values of ECD(F:A) and ECD(F:I) as compared to ECD(I:A) and ECD(I:I).

**Figure 4:**
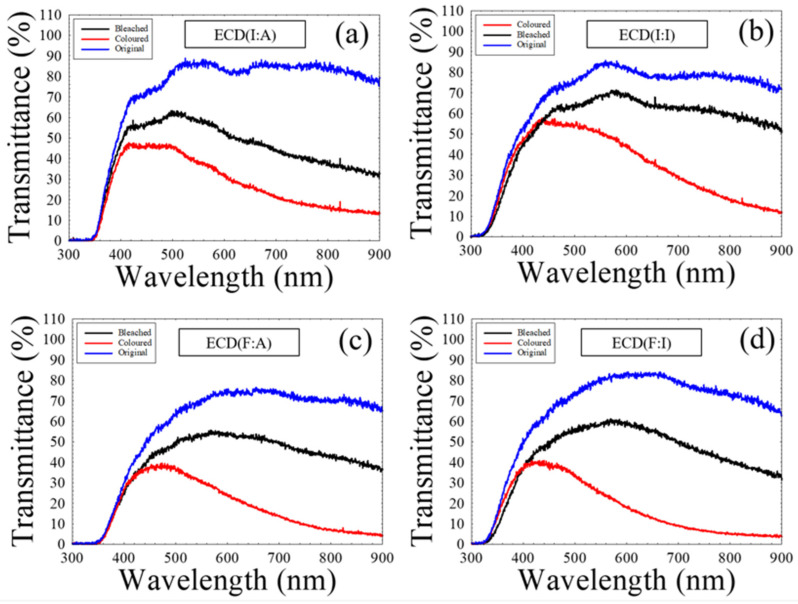
The original, coloring, and bleaching transmittances of (**a**) ECD(I:A), (**b**) ECD(I:I), (**c**) ECD(F:A), and (**d**) ECD(F:I).

In terms of CA measurements, the study of switching characteristics was investigated for all the ECDs. The ECDs were measured at a sweeping voltage from −3 V to 3 V, while the coloring and bleaching transmittance was recorded simultaneously. Figure 4 represents the original, coloring, and bleaching transmittances of all the ECDs. Before any measurements, these ECDs are highly transparent with high optical transmittance values between 80 to 90% in the visible wavelength region. Following the completion of CA measurements, the ECD(F:I) acquired the most considerable optical modulation of 42% with a coloring transmittance of 14% and a bleaching transmittance of 56%. In contrast, a relatively large optical modulation of 31% with 52% bleaching transmittance and 21% coloring transmittance was observed for ECD(F:A). For ECD(I:I), the device reported a 64% bleaching transmittance and 38% coloring transmittance with an optical modulation of 26%. Among all the ECDs, the lowest optical modulation of 21% with 49% in the bleached state and 28% in the colored state was measured with ECD(I:A). It should be noted that due to the dark blue color in the colored state of the ECD, it should be logical that the transmittance is higher around the blue region (400–450 nm). On the other hand, transmittance decreases when approaching the infrared region since ECDs are designed to block the red region (600 nm onwards) and infrared rays from incident light.

In this study, the coloration efficiency (CE) was calculated, which is defined as the ratio between the change in optical density and inserted charge per unit area. The CE is known as an essential aspect of EC study, which indicates the performance of ECDs. Therefore, it is evaluated based on the formula below [21]:

(3) $$\mathrm{CE}=\frac{\Delta OD}{Q_{in}}=\frac{\log\left(\frac{T_{b}}{T_{c}}\right)\;}{Q_{in}}$$

where Δ*OD* is the optical density, *Q_in_* is the inserted charge, *T_b_* is the bleaching transmittance, and *T_c_* is the coloring transmittance. Based on the equation, the coloration efficiency and charge were tabulated in Table 2.

**Figure 5:**
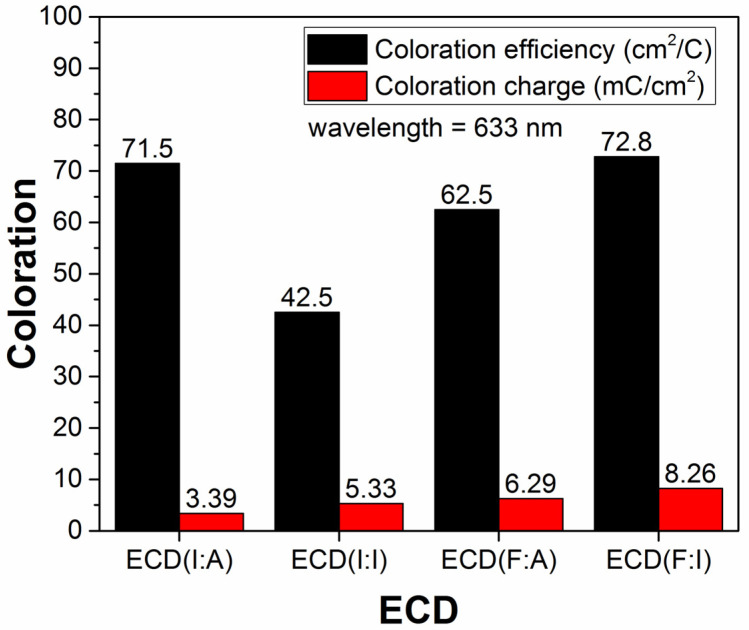
The coloration efficiency and charge of ECD(I:A), ECD(I:I), ECD(F:A), and ECD(F:I) devices.

At the wavelength value of 633 nm in Figure 5, the CE for ECD(F:I) of 72.8 cm^2^/C was the highest, followed by 71.5 cm^2^/C for ECD(I:A), 62.5 cm^2^/C for ECD(F:A), and 42.5 cm^2^/C for ECD(I:I). It was proposed that the high CE value obtained in ECD(F:I) was likely due to the large optical modulation of the device. In contrast, despite ECD(I:A) having low optical modulation, the high CE calculated can be attributed to the low intercalated charge. The coloration charge in ECDs with AZO as the counter electrode was lower when compared to the ECDs with ITO as the counter electrode. In comparison, ECD(I:A) produced a coloring charge of 3.39 mC/cm^2^, ECD(I:I) produced 5.33 mC/cm^2^, ECD(F:A) produced 6.29 mC/cm^2^, and ECD(F:I) produced 8.26 mC/cm^2^. On top of that, several reports demonstrated that FTO and ITO have superior electrical properties as compared to AZO [31,32,33,34]. In the work of Yilei et al., they revealed the lowest resistivity of AZO to be in the range of 10^−2^ Ω cm, while the lowest resistivity of ITO was in the range of 10^−4^ Ω cm. The authors further explained that the difference in electrical properties was ascribable to the smaller grain size and strip-shaped grains [31]. Hence, this further confirms the observation of the ECDS in this study.

### 3.3. EC Mechanism of WO_3_-Based ECDs with Conducting Polymer Electrolyte

**Figure 6:**
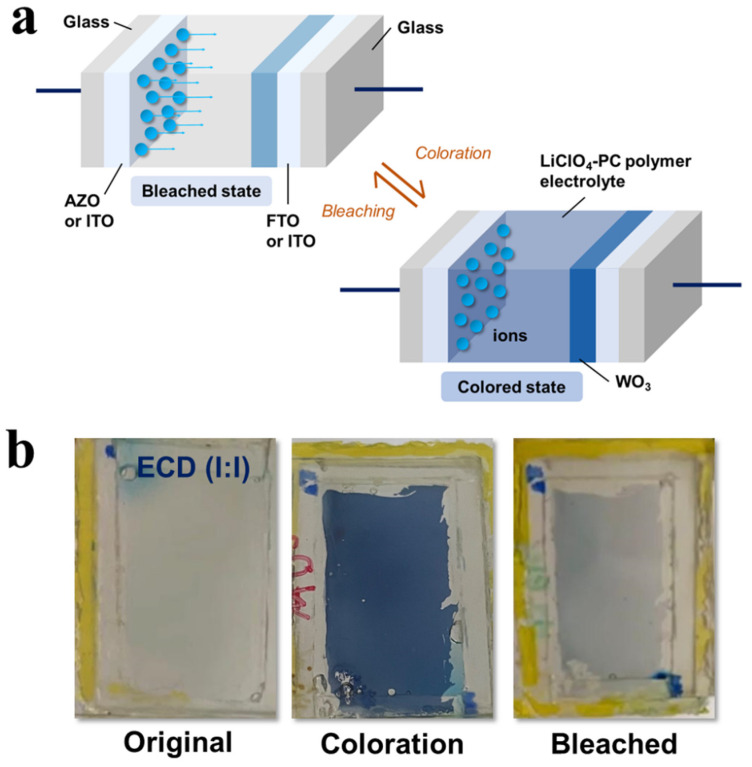
(**a**) The schematic coloration and bleaching processes of ECD(I:A), ECD(I:I), ECD(F:A), and ECD(F:I) devices. (**b**) The photographs of the actual EC device (ECD(I:I)) in the original, coloring, and bleaching states as examples in this study.

In this study, the conducting LiClO_4_-PC polymer electrolyte was utilized to transport the ions between the anodes and cathodes for the ECD coloration in bleaching processes (see Figure 6). When a potential voltage was applied across the ECD, the process caused a change in the oxidation state of W from 6+ to 5+ [35]. Coloration develops as ions are introduced into the WO_3_ films from LiClO_4_-PC, which combines with electrons provided by the external source. Alternatively, the bleaching process occurs when an opposite potential is applied to remove the ions, causing the ECD to become transparent. Based on the various ECDs in this study, the mechanisms of the coloration and bleaching process were still similar through the conducting polymer electrolyte regardless of the choices in TCOs or the counter electrodes. Nonetheless, the performance of the ECDs depends on the diffusion efficiencies as different substrates were used. As a result, the optimization process showed that, although FTO might be advantageous, the corresponding counter electrodes would need to match the overall design structure of the ECDs.

## 4. Conclusions

Conclusively, the WO_3_ films were fabricated by using the sol–gel and spin-coating method on ITO- and FTO-coated substrates. In ECD(F:A), the CV results revealed the highest cathodic peak current and, subsequently, the most significant diffusion coefficient. Nevertheless, the smaller diffusion coefficient of ECD(I:A) and ECD(I:I) was due to the increase in sheet resistivity of ITO during the post-annealing process. The ECD(F:I) exhibited the largest CE of 72.53 cm^2^/C with a sizeable optical modulation of 42%. Furthermore, the lower coloration charge observed in ECD(I:A) and ECD(F:A) was due to the inferior electrical properties in AZO films as compared to ITO and FTO. Resultantly, a basic ECD should possess a balance optimization process among important EC components. While a high CE value is desired, the ECD should also exhibit a large optical modulation and good ion storage capacity. Although AZO exhibited inferior electrical properties, they offered more outstanding CE due to reduced charges needed for the coloration process. Through this study, the role of TCOs in WO_3_-based ECDs with conducting polymer electrolytes was elaborated. Hence, the optimization process of WO_3_-based ECDs can be achieved for the rapid development of EC technology. This work demonstrated the feasibility to replace ITO and FTO with AZO as counter electrodes, thus reducing the fabrication cost due to the high cost of indium. This is in line with the industrial trend of deploying more ZnO and doped counterparts in optoelectronic applications due to better transparency and lower material cost, in addition to an environmentally safe material system.

## Figures and Tables

**Table 1:** Summary of the calculated diffusion coefficient values of the ECDs with various TCOs.

ECD	Anodic Peak Current, mA	Cathodic Peak Current, mA	Anodic Diffusion Coefficient, cm^2^/s^−1^	Cathodic Diffusion Coefficient, cm^2^/s^−1^
ECD(I:A)	0.164	−0.662	2.59 × 10^−13^	4.21 × 10^−12^
ECD(I:I)	0.443	−1.142	1.89 × 10^−12^	1.25 × 10^−11^
ECD(F:A)	0.834	−1.705	6.69 × 10^−12^	2.79 × 10^−11^
ECD(F:I)	0.706	−1.665	4.79 × 10^−12^	2.66 × 10^−11^

**Table 2:** Summary of the calculated coloration efficiency and charge values of the ECDs with various TCOs.

ECD	Coloration Efficiency (cm^2^/C)	Coloration Charge (mC/cm^2^)
ECD(I:A)	71.5	3.39
ECD(I:I)	42.5	5.33
ECD(F:A)	62.5	6.29
ECD(F:I)	72.8	8.26

## Data Availability

Not applicable.
